# Comparison of rectal and bladder dose between retractor insertion and gauze packing in intracavitary brachytherapy for cervical cancer

**DOI:** 10.1093/jrr/rraf042

**Published:** 2025-08-06

**Authors:** Noriko Osaki, Takashi Soyano, Shinya Sutani, Hideki Matsumoto, Atsuya Takeda, Atsunori Yorozu

**Affiliations:** Department of Radiation Oncology, Saiseikai Yokohamashi Tobu Hospital, 3-6-1, Shimosueyoshi, Tsurumi-ku, Yokohama, Kanagawa 230-8765, Japan; Department of Radiation Oncology, National Hospital Organization Tokyo Medical Center, 2-5-1 Higashigaoka, Meguro-ku, Tokyo 152-0021, Japan; Department of Radiation Oncology, National Hospital Organization Tokyo Medical Center, 2-5-1 Higashigaoka, Meguro-ku, Tokyo 152-0021, Japan; Department of Radiation Oncology, National Hospital Organization Tokyo Medical Center, 2-5-1 Higashigaoka, Meguro-ku, Tokyo 152-0021, Japan; Department of Radiation Oncology, National Hospital Organization Tokyo Medical Center, 2-5-1 Higashigaoka, Meguro-ku, Tokyo 152-0021, Japan; Department of Radiology, Keio University School of Medicine, Shinanomachi 35, Shinjuku-ku, Tokyo 160-8582, Japan; Department of Radiation Oncology, National Hospital Organization Tokyo Medical Center, 2-5-1 Higashigaoka, Meguro-ku, Tokyo 152-0021, Japan

**Keywords:** cervical cancer, intracavitary brachytherapy, gauze packing, rectal retractor

## Abstract

This study aimed to compare and verify the rectal and bladder doses of intracavitary brachytherapy (ICBT) using both rectal retractor (RR) and gauze packing (GP) in the same patients. A total of 37 patients who underwent ICBT using RR and GP for cervical cancer were included in this study. Rectal and bladder dose and volume data were compared with the RR and GP treatments in the same patients and the confounding factors were examined. When comparing RR and GP, the median and interquartile ranges for rectal D2cc were 2.8 (2.5–3.7) Gy with RR and 3.2 (2.7–3.8) Gy with GP. The median bladder D2cc was 4.9 (4.5–6.3) Gy with RR and 4.8 (3.9–5.4) Gy with GP. The Wilcoxon signed-rank test revealed that rectal doses were significantly lower with RR (*P* = 0.02), whereas bladder doses were significantly higher with RR (*P* < 0.001). Analysis of the correlation between the number of gauze pieces and the difference in rectal D2cc between GP and RR using Pearson’s distribution revealed no significant correlation (*R* = −0.20, *P* = 0.22), as well as bladder D2cc between GP and RR also revealed no significant correlation (*R* = −0.20, *P* = 0.22). The number of gauze pieces did not necessarily correlate with a reduction in the rectal and bladder dose. In conclusion, rectal D2cc was lower with RR in image-guided brachytherapy for cervical cancer, whereas bladder D2cc was higher with RR than with GP.

## INTRODUCTION

The standard treatment for definitive radiotherapy of cervical cancer is a combination of external beam radiation therapy (EBRT) and intracavitary brachytherapy (ICBT) [1]. In combination therapy, late complications such as proctitis and rectal bleeding, as well as cystitis and bladder bleeding, are significant issues; reported incidences range from 5% to 52.2% for rectal toxicity and 23.4%–31% for bladder toxicity [2–11]. To reduce these adverse effects, the radiation dose to these organs must be decreased [4]. Common methods to reduce the rectal ICBT dose include gauze packing (GP), in which several pieces of gauze are inserted around the applicator in the vagina to secure it and reduce the rectal dose; using a thin, flat rectal retractor (RR) that can be fixed to the tandem and placed posterior to the ovoids; and inserting cylindrical spacers that cover the applicator, and inflating a balloon within the vagina. To decrease the bladder dose, methods such as the use of larger ovoids and tight GP from the vaginal fornix to the anterior vaginal wall are employed. GP is an older and more widely used technique. However, in recent years, RRs manufactured by medical device companies have become increasingly available. In comparison to GP, which requires meticulous insertion of gauze without gaps, RR reduces procedure time and minimizes operator variability. This study aimed to compare and verify the rectal and bladder doses of ICBT using both RR and GP in the same patients.

## MATERIALS AND METHODS

### Patients

A retrospective analysis was conducted on patients aged 20–89 years who underwent ICBT for cervical cancer at our institution between March 2017 and December 2021. Patients who underwent both GP and RR placement using a plastic applicator were included. The applicator was a tandem and ovoid type with three source lines. Patients treated with interstitial brachytherapy were excluded.

### Treatment

Since 2001, EBRT (40–50.4 Gy) with and without central shielding has been followed by four sessions of ICBT over 8–10 days at the National Hospital Organization Tokyo Medical Center. Traditionally, ICBT has been performed using a metal applicator (Fletcher-Williamson Applicator, Elekta) and GP to reduce the rectal dose. Since the introduction of magnetic resonance imaging (MRI)-based image-guided brachytherapy (IGBT) in March 2017, a plastic applicator (Fletcher CT/MRI Applicator, Elekta) has been used for intraoperative MRI. This plastic applicator is equipped with an RR that can be used whenever it is insertable.

Due to the limited number of available applicators, MRI-based treatment planning is typically conducted only for the first session. The RR was available in two sizes: large (4 cm wide, 4 mm thick) and small (3 cm wide, 4 mm thick); an appropriate size was selected for each patient. The remaining three sessions were planned using computed tomography (CT) with a metal applicator. GP was performed around the applicator on both the rectal and bladder sides, with the number of gauze pieces adjusted according to the patient’s vaginal width. The size of the gauze was 7.5 × 15 cm. Depending on the presence of pain or the operator’s skills, adequate insertion of the gauze may be difficult. In each case, multiple doctors reviewed the CT images to ensure appropriate gauze insertion. The initial MRI images were fused with CT for treatment planning, and high-risk clinical target volume (HR-CTV) was delineated according to The Groupe Européen de Curiethérapie and the European Society for Radiotherapy & Oncology (GEC-ESTRO) recommendations [12]. The treatment dose was calculated based on the Manchester method using the same loading pattern for all sessions. Treatment planning was performed using Oncentra Brachy software (Ver. 4.5.4, Elekta). Rectal and bladder dose and volume data were collected for both the RR and GP treatments in the same patients.

### Data analysis

To ensure consistency in comparing treatments within the same patient, the ovoid size, tandem size and the widths of ovoid spacing were kept the same. The ovoid height was 30 mm, with plastic applicators having a width of 20 mm, corresponding to metal applicators categorized as SS (20 mm), S (25–30 mm) and M (35–40 mm). Each patient typically received three GP treatments; however, for analysis, data from the first RR treatment and the corresponding GP treatment using the same applicator size were extracted and evaluated.

Because the prescribed doses varied between treatments, even for the same patient, the minimum dose delivered to 90% of the target volume (D90) of the HR-CTV as converted to an equivalent dose of 6 Gy. Subsequently, the minimum dose delivered to 2 cc of the target volume (D2cc) doses to the rectum, bladder and sigmoid colon were compared between RR and GP plans for the same patient. The cumulative dose from EBRT and ICBT was calculated based on GEC-ESTRO recommendations [12]. For cases involving central shielding, the HDR radiobiologic dose-equivalent worksheets from the American Brachytherapy Society were used for dose summation. For a single dose of 1.8 Gy with central shielding, the tumor received an additional 0.8 Gy (*α*/*β* = 10) and the organs at risk (OAR) received an additional 0.7 Gy (*α*/*β* = 3) [13].

The Wilcoxon signed-rank test was used to compare doses between the two groups (RR and GP). We hypothesized that the difference in rectal and bladder doses between GP and RR would decrease as the number of gauze pieces increased. The correlation between gauze quantity and dose difference between the RR and GP in the rectum and bladder was analyzed using Pearson’s correlation. The Waterfall plot was created about the difference in rectal D2cc between GP and RR (RD2cc GP–RR) and the difference in bladder D2cc between GP and RR (BD2cc GP–RR). Confounding factors that might influence the relationship between rectal D2cc and RR or GP were also considered, including differences in ovoid size between plastic and metal applicators (SS, S and M), age, parity and body mass index (BMI). Acute adverse events were defined as those occurring within 90 days after the radiotherapy and late adverse events as those occurring after this period.

In all statistical tests, a *P*-value <0.05 was considered statistically significant. Statistical analyses were performed using JMP software (ver. 15). Fisher’s exact test was used to analyze the differences in ovoid size, whereas other comparisons were made using the Wilcoxon signed-rank test.

## RESULTS

**Fig. 1:**
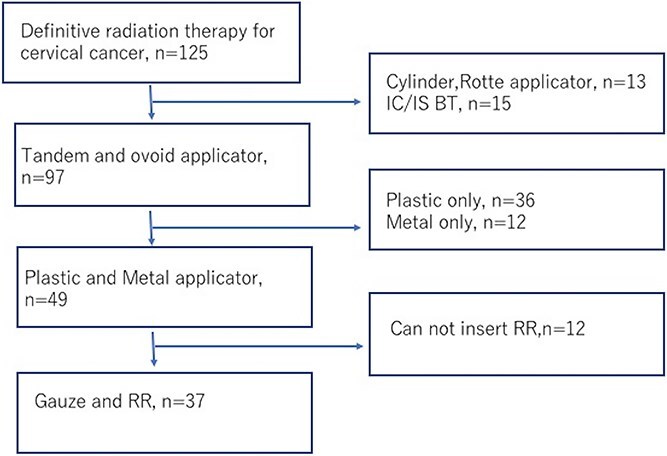
Patient selection. IC/IS BT, intracavitary and interstitial brachytherapy; RR, rectal retractor.

A total of 37 patients who underwent ICBT using RR and GP for cervical cancer between March 2017 and November 2021 were included (Fig. 1). Table 1 shows the characteristics of the patient. The median prescribed dose to point A was 4.5 Gy (interquartile range, IQR: 4–4.5 Gy), and the median HR-CTV D90 was 5.7 Gy (IQR: 5.2–6.6 Gy) before conversion. All patients received a combination of EBRT with a total dose of 45–50.4 Gy. Irradiation methods included intensity-modulated radiation therapy in 17 patients and three-dimensional conformal radiation therapy in 20 (16 of which involved central shielding).

**Table 1:** Patient characteristics

Characteristic	
Age [IQR]	53 [45–69]
BMI [IQR]	20.0 [17.9–24.1]
T stage	
T1b	4
T2a	2
T2b	16
T3a	2
T3b	13
Delivery history	
+	26
−	11
Ovoid size	
SS	12
S	47
M	15
RR	
Small	29
Large	8
Gauze [IQR]	11 [5–18]

IQR, interquartile range; RR, rectal retractor.

The median cumulative dose combining EBRT and ICBT was as follows: CTV HR D90: 76.6 Gy (IQR: 73.5–80.7 Gy), rectal D2cc: 61.3 Gy (IQR: 57.8–63.5 Gy), bladder D2cc: 73.7 Gy (IQR: 69.6–77.9 Gy) and sigmoid colon D2cc: 60.2 Gy (IQR: 56.6–62.3 Gy) (Table 2). When comparing RR and GP, the median and IQRs for rectal D2cc were 2.8 (2.5–3.7) Gy with RR and 3.2 (2.7–3.8) Gy with GP. The median bladder D2cc was 4.9 (4.5–6.3) Gy with RR and 4.8 (3.9–5.4) Gy with GP. The median sigmoid colon D2cc was 3.0 (2.1–3.9) Gy with RR and 2.9 (2.4–3.8) Gy with GP (Table 3). The Wilcoxon signed-rank test revealed that rectal doses were significantly lower with RR (*P* = 0.02), bladder doses were significantly higher with RR (*P* < 0.001), and there was no significant difference in the sigmoid colon doses (*P* = 0.25).

**Table 2:** The median total doses to the OAR and target

	EBRT+RALS
CTV HR D90 median [IQR]	76.6 [73.5–80.7] Gy
Rectum D2cc median [IQR]	61.3 [57.8–63.5] Gy
Bladder D2cc median [IQR]	73.7 [69.6–77.9] Gy
Sigmoid D2cc median [IQR]	60.2 [56.6–62.3] Gy

OAR, organ at risk; IQR, interquartile range.

**Table 3:** The median dose to the OAR at the RR and GP

	RR	GP
Rectum D2cc median [IQR]	2.8 [2.5–3.7] Gy	3.2 [2.7–3.8] Gy
Bladder D2cc median [IQR]	4.9 [4.5–6.3] Gy	4.8 [3.9–5.4] Gy
Sigmoid D2cc median [IQR]	3.0 [2.1–3.9] Gy	2.9 [2.4–3.8] Gy

OAR, organ at risk; RR, rectal retractor; GP, gauze packing; IQR, interquartile range.

**Fig. 2:**
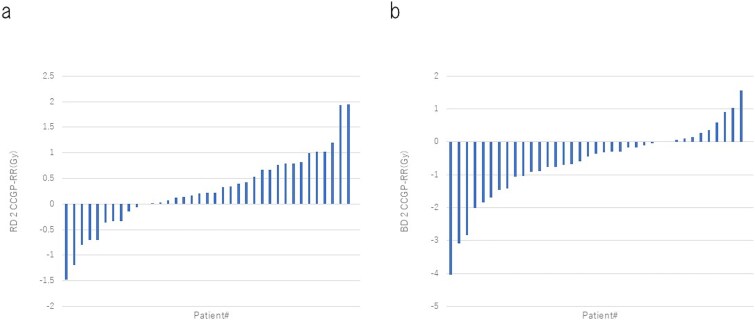
(a) D2cc of the rectum of individuals. RD2cc, D2cc of rectum; RR, rectal retractor; GP, gauze packing. (b) D2cc of the bladder for each individual. BD2cc, D2cc of the bladder; RR, rectal retractor; GP, gauze packing.

A waterfall plot of the difference in RD2cc GP–RR showed that 73% of the patients had RD2cc GP–RR > 0, indicating a higher rectal dose with GP (Fig. 2a). Similarly, a waterfall plot of the difference in BD2cc GP–RR showed that 70% of the patients had a BD2cc GP–RR < 0, indicating a higher bladder dose with RR (Fig. 2b). Analysis of the correlation between the number of gauze pieces and RD2cc GP–RR using Pearson’s distribution revealed no significant correlation (*R* = −0.20, *P* = 0.22). Similarly, analysis of the correlation between the number of gauze pieces and BD2cc GP–RR also revealed no significant correlation (*R* = −0.20, *P* = 0.22).

The median RD2cc GP–RR was 0.22, and patients were divided into low (RD2cc GP–RR < 0.22) and high (RD2cc GP–RR ≥ 0.22) groups. Wilcoxon two-sample tests revealed no significant differences in age (*P* = 0.92), number of gauze pieces (*P* = 0.36) or BMI (*P* = 0.22) between the groups. Fisher’s exact test revealed no significant differences in delivery (*P* = 0.73) or ovoid size (SS/S: *P* = 0.22; S/M: *P* = 0.63; SS/M: *P* = 1.00) (Table 4). The median BD2cc GP–RR was −0.35, and patients were divided into low (BD2cc GP–RR < −0.35) and high (BD2cc GP–RR ≥ −0.35) groups. Wilcoxon two-sample tests revealed no significant differences in age (*P* = 0.68), number of gauze pieces (*P* = 0.70) or BMI (*P* = 0.55) between the groups. Fisher’s exact test revealed no significant differences in delivery (*P* = 0.71) or ovoid size (SS/S: *P* = 1.00; S/M: *P* = 1.00; SS/M: *P* = 1.00) (Table 5).

**Table 4:** Comparison of patient backgrounds between the low and high RD2cc GP–RR groups

	Low, *n* = 18	High, *n* = 19	*P* value
Age [IQR]	52.5 [47–70.25]	55 [43–69]	*P* = 0.92
Number of gauzes [IQR]	12 [10.25–15.25]	10 [8–13]	*P* = 0.36
Delivery history			
+	12	14	*P* = 0.73
−	6	5	
Size of ovoid			
SS	2	6	SS/S *P* = 0.22
S	14	10	S/M *P* = 0.63
M	2	3	SS/M *P* = 1.00
BMI	22.3 [17.7–25.9] (*n* = 17)	19.3 [17.6–22.9] (*n* = 19)	*P* = 0.22

RR, rectal retractor; GP, gauze packing; RD2cc GP–RR, rectal D2cc between GP and RR; RD2cc, D2cc of rectum; IQR, interquartile range.

**Table 5:** Comparison of patient backgrounds between the low and high BD2cc GP–RR groups

	Low, *n* = 18	High, *n* = 19	*P* value
Age [IQR]	51 [43–72]	55 [50–69]	*P* = 0.68
Number of gauzes [IQR]	11 [9–14]	12 [8–15]	*P* = 0.70
Delivery history			
+	14	12	*P* = 0.48
−	4	7	
Size of ovoid			
SS	4	4	SS/S *P* = 1.00
S	12	12	S/M *P* = 1.00
M	2	3	SS/M *P* = 1.00
BMI	20.1 [17.2–25.6] (*n* = 17)	19.9 [17.8–22.7] (*n* = 19)	*P* = 0.55

RR, rectal retractor; GP, gauze packing; BD2cc GP–RR, bladder D2cc between GP and RR; BD2cc, D2cc of bladder; IQR, interquartile range.

Of the 37 patients, 26 had a history of delivery. The median age of patients with a history of delivery was 64.5 years, whereas that of those without a history of delivery was 45 years (*P* < 0.001). The median number of gauze pieces was 11 in patients with a history of delivery and 12 in those without, showing no significant difference (*P* = 1.00). The median RD2cc GP–RR was 0.28 in patients with a history of delivery and 0.21 in those without, showing no significant difference (*P* = 0.48). The median BD2cc GP–RR was −0.51 in patients with a history of childbirth and −0.18 in those without, showing no significant difference (*P* = 0.53). When the patients were divided into two groups based on age, 21 patients were under 60 years old and 16 were 60 years or older. The median number of gauze pieces was 13 in patients under 60 and 9 in those 60 years or older (*P* < 0.001). The median RD2cc GP–RR was 0.16 in patients under 60 and 0.28 in those 60 years or older, with no significant difference (*P* = 0.074). The median BD2cc GP–RR was −0.44 in patients under 60 and −0.34 in those 60 years or older, with no significant difference (*P* = 0.56). Pearson’s analysis showed no correlation between BMI and the number of gauze pieces (*R* = −0.25, *P* = 0.14).

Regarding intraoperative adverse events, two cases of vaginal lacerations were observed with both GP and RR, but were minor and resolved with pressure hemostasis. Two late adverse events were observed: one patient experienced rectal bleeding and underwent Argon Plasma Coagulation (APC), and the other experienced both rectal and bladder bleeding simultaneously, requiring APC and blood transfusion. Both cases involved significant interventions corresponding to grade 3 on the CTCAE v5.0; however, there were no grade 4 or higher events [14].

## DISCUSSION

In this study, an analysis of 37 patients revealed that significantly more patients had lower rectal D2cc doses when treated with RR than when treated with GP. However, the bladder D2cc doses were significantly higher in patients treated with RR than in those treated with GP. According to a report by Kong *et al.*, a comparison of RR and GP in the same patients showed that rectal and sigmoid colon D2cc were significantly lower with RR, whereas bladder doses were not significant. Our study supports the finding that RR can effectively reduce rectal D2cc [15].

**Fig. 3:**
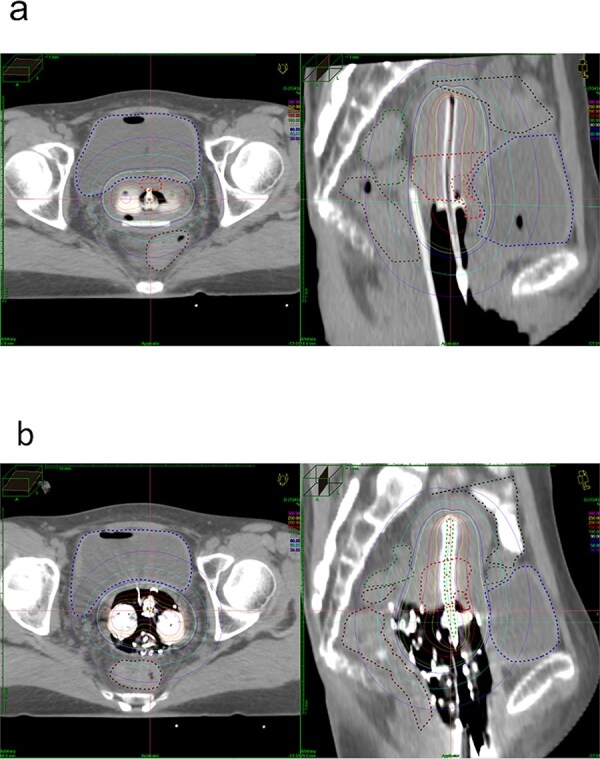
Axial and sagittal of computed tomography (CT) images of the same patient. (a) CT images taken during treatment with RR (RD2cc: 3.3 Gy, BD2cc: 5.8 Gy). (b) CT images taken during treatment with GP (RD2cc: 3.8 Gy, BD2cc: 3.3 Gy). RD2cc, D2cc of rectum; BD2cc, D2cc of the bladder; RR, rectal retractor; GP, gauze packing.

As shown in Fig. 3a, RR can uniformly move the rectum away from the applicator; however, it does not adequately move the bladder wall away from the applicator. On the other hand, even if sufficient gauze appears to be packed, it is often not as effective in reducing the rectal dose as is RR. However, gauze is effective in separating the bladder wall from the applicator, as shown in Fig. 3b. During treating with RR, it is thought that the bladder dose can be reduced by packing the ventral side of the applicator with gauze; this is a topic for future study. Unfortunately, this study did not involve cases with simultaneous use of both GP and RR, therefore, this is a discussion pertains to cases treated with RR alone.

Recently, Sawada *et al.* conducted a similar study comparing GP and RR in the same patients and reported that GP reduced rectal doses more effectively than RR. However, our study found that the number of gauze pieces did not necessarily correlate with a reduction in the rectal dose. This suggests that proper placement techniques, such as careful insertion of gauze along the anterior–posterior axis of the ovoid, and the effectiveness of GP depend heavily on the operator’s skill [16]. Additionally, RRs can be challenging to insert into patients with a narrow vaginal introitus, even if the vaginal fornix is spacious. In patients with a narrow vaginal introitus and wide fornix, GP might be more effective at reducing rectal doses around the flange of the tandem, highlighting the influence of patient anatomy.

Sawada *et al.* reported that patients with a low BMI, due to a lack of perineal fat and a narrower vaginal fornix, experienced greater rectal dose reduction with GP. In our study, BMI was not identified as a confounding factor for RD2cc GP–RR. Additionally, the mean BMI of our study and that of Sawada *et al.* are both similar. The differences in results between these two studies may be largely attributable to variations in patient anatomy and operator skill and also indicate that evaluation of patient anatomy using BMI alone may be insufficient.

No confounding factors were identified for RD2cc GP–RR. While not statistically significant, patients aged 60 and older tended to benefit more from rectal dose reduction with RR compared to GP. This was probably because the number of gauze pieces that could be inserted in the elderly was not sufficient to reduce the rectal dose. Yanazume *et al.* reported that older patients experience reduced vaginal elasticity and moisture owing to aging, making ICBT inoperable for patients without a history of vaginal childbirth [17]. In this study, delivery did not affect the ovoid size or number of gauze pieces; however, the younger age of patients without delivery may have influenced these results. Despite a significant difference in gauze quantities, there was no significant difference between the group under 60 years old and the group 60 years and older in BD2cc GP–RR; it is possible that additional gauze was placed posteriorly.

GP may reduce the bladder dose as gauze is packed on the anterior side, which is not achieved with RR. The ring-type applicator used in the study by Kong *et al.* had an anterior component around the tandem, allowing for bladder dose reduction even without GP. In the future treatment of RR, strategies such as hybrid applicators or combining interstitial therapy to reduce anterior doses should be considered. The median cumulative CTV HR D90 for EBRT and ICBT was 76.6 Gy, which was lower than the target set by the EMBRACE II study [18]. This is comparable to the findings of a report by Toita *et al.*, which focused on Japanese patients [19].

Vaginal lacerations occurred in two cases each for RR and GP during treatment. Gupta *et al.* reported a 0.11% incidence of vaginal lacerations in patients with GP, which was lower than that reported in this study. However, all cases were minor and resolved with pressure hemostasis, with no significant increase in the incidence associated with RR [20]. The late complications in this study were comparable to the findings of the EMBRACE II study, which reported grade 3 complications in 2% of rectal and 6% of bladder cases [16]. Currently, new applicators, combined interstitial therapy and dose optimization methods are emerging as strategies for reducing bladder and rectal doses. It is important to consider the patient’s body type and treatment method when selecting an appropriate treatment approach.

This study had several limitations. Patients with narrow vaginal canals who could not accommodate a Fletcher applicator, as well as those in whom the Fletcher applicator could be inserted but the RR could not, were excluded. Additionally, although the ovoids used were from the same manufacturer and labeled similarly, there were slight differences in shape between the metal and plastic ovoids; this could have influenced the vaginal extension and, subsequently, rectal doses. The sample size was also insufficient to detect statistical significance in some cases, particularly among older patients without a history of childbirth, which may not have been identified as a confounding factor. Although the number of gauze pieces could be quantified, the specific techniques used by the operator could not, making it difficult to assess the impact of these techniques; further research is needed to determine which patients benefit the most from RR. Lastly, although the RR was faster to insert and remove, even under sedation, and resulted in fewer complaints of discomfort, no objective measure of the insertion time was collected. In the future, it will be important to objectively assess the simplicity of RR insertion. Despite these limitations, this study is a valuable study showing that RR reduces rectal dose compared to GP in the same patient.

In summary, RR can reduce rectal doses more than GP. However, the RR could not be inserted in 12 patients, indicating that this method is not always suitable. GP, on the other hand, can be adjusted by varying the number of gauze pieces, making it adaptable for patients with a narrow vaginal canal. In conclusion, rectal D2cc was lower with RR in IGBT for cervical cancer, whereas bladder D2cc was higher with RR than with GP.
